# Supplementary material for: Human immune and gut microbial parameters associated with inter-individual variations in COVID-19 mRNA vaccine-induced immunity
Source: Commun Biol. 2023 Apr 20;6:368. doi: 10.1038/s42003-023-04755-9 (PMC10119155; doi:10.1038/s42003-023-04755-9)
Supplement: Supplementary file 2 — Supplementary Information [file 42003_2023_4755_MOESM2_ESM.pdf]

Supplementary Figure 1

a

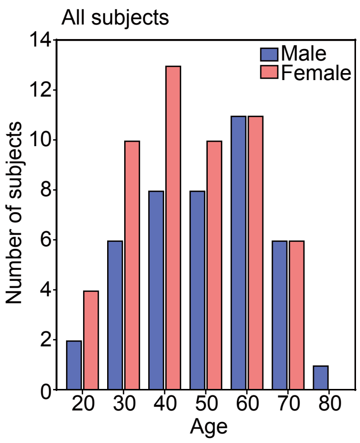

b

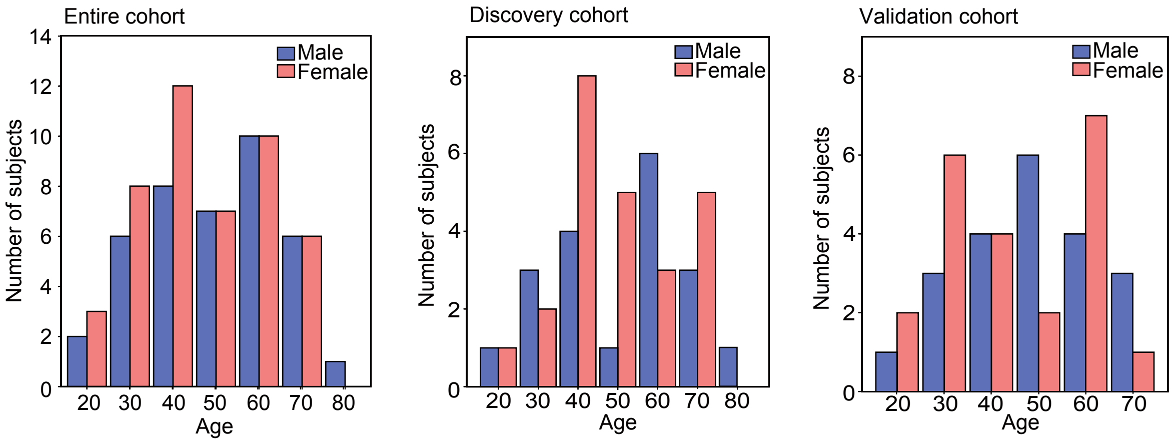

Supplementary Fig. 1. Age and sex distribution of study participants.

(a, b) Age and sex distribution in 96 participants **(a)**, entire, discovery, and validation cohorts **(b).**

Supplementary Figure 2

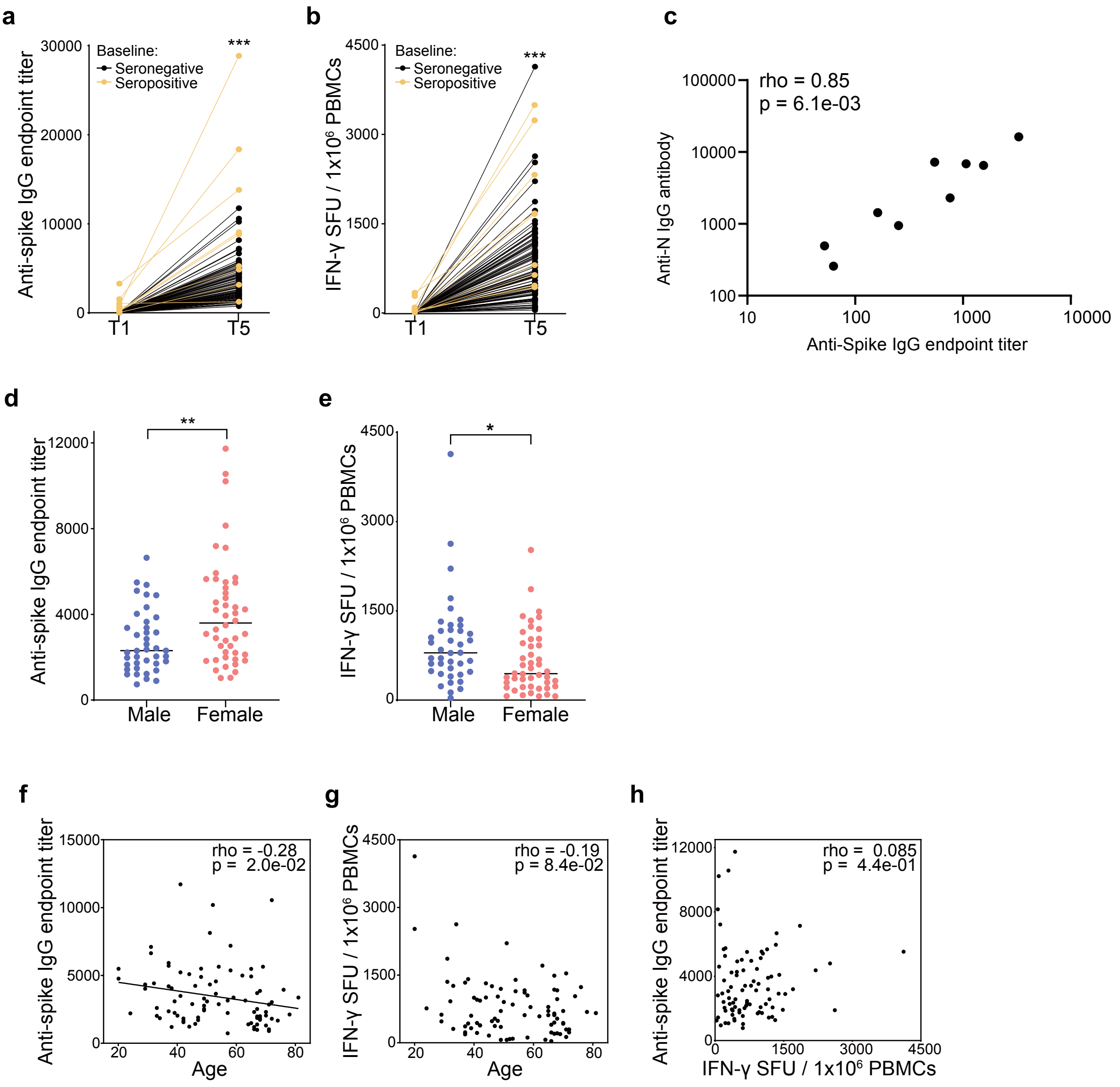

Supplementary Fig. 2. Inter-individual variations in BNT162b2-induced adaptive immunity.
(a) Anti-SARS-CoV-2 spike IgG endpoint titers in plasma at T1 and T5 were measured using ELISA (n = 95). **(b)** IFN-γ-secreting T cells specific for SARS-CoV-2 spike in PBMCs at T1 and T5 were measured with ELISpot assays (n = 95). SFU, spot-forming unit. **(c)** Correlation between levels of baseline antibodies specific to SARS-CoV-2 spike and N antigens. Subjects who were seropositive for SARS-CoV-2 spike (n = 9) at T1 were subjected to SARS-CoV-2 N antibody test using serum collected at T1. **(d, e)** BNT162b2-induced antibody responses (anti-SARS-CoV-2 spike IgG endpoint titers at T5) **(d)** and T-cell responses (IFN-γ-secreting T cells specific for SARS-CoV-2 spike in PBMCs at T5) **(e)** in male (n = 40) and female (n = 46) subjects seronegative for SARS-CoV-2 spike. **(a, b, d, e)** p values were calculated with Wilcoxon rank-sum tests (* $p < 0.05$, ** $p < 0.01$, *** $p < 0.001$). **(f, g)** Correlation analysis between age and BNT162b2-induced antibody responses **(f)** or T-cell responses **(g)**. **(h)** Correlation analysis between BNT162b2-induced antibody responses and T-cell responses. **(c, f-h)** Correlations were analyzed with Spearman's correlation. p values were corrected with Benjamini–Hochberg FDR correction for multiple tests. Spearman's rho coefficient and p values are indicated in the plots.

Supplementary Figure 3

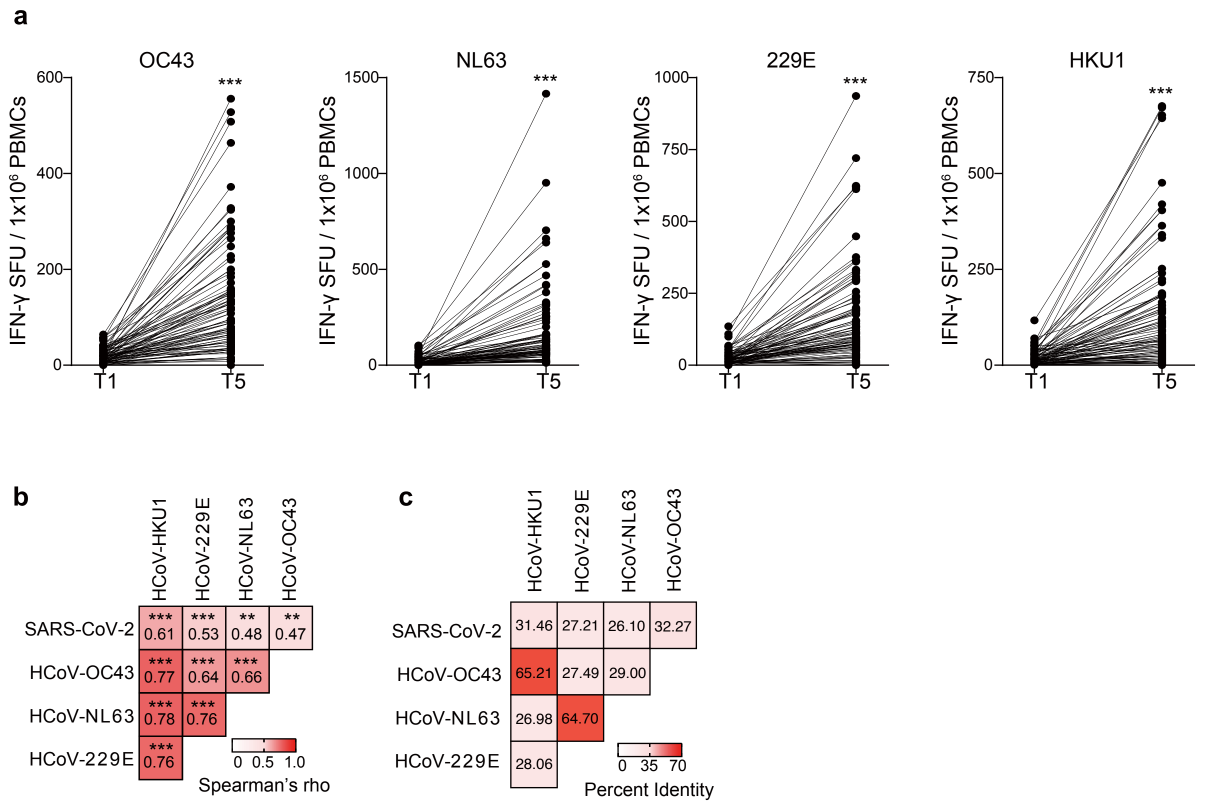

Supplementary Fig. 3. BNT162b2-induced T-cell responses against human common cold coronaviruses.
(a) IFN-γ-secreting T cells specific for spike of HCoV-OC43, NL63, 229E, and HKU1 in PBMCs at T1 and T5 were measured with ELISpot assays (n = 86). SFU, spot-forming unit. p values were calculated by Wilcoxon rank-sum tests (*** $p < 0.001$). **(b)** Heat map showing correlations between vaccine-induced T cell responses against SARS-CoV-2 and HCoVs. Correlations were analyzed with Spearman's correlation. p values were corrected with Benjamini–Hochberg FDR correction for multiple tests. Spearman's rho coefficient and p values are indicated in heat map cells (** $p < 0.01$, *** $p < 0.001$). **(c)** Heat map showing percentages of amino acid sequence identify for spike proteins from SARS-CoV-2 and HCoVs analyzed using Clustal Omega.

Supplementary Figure 4

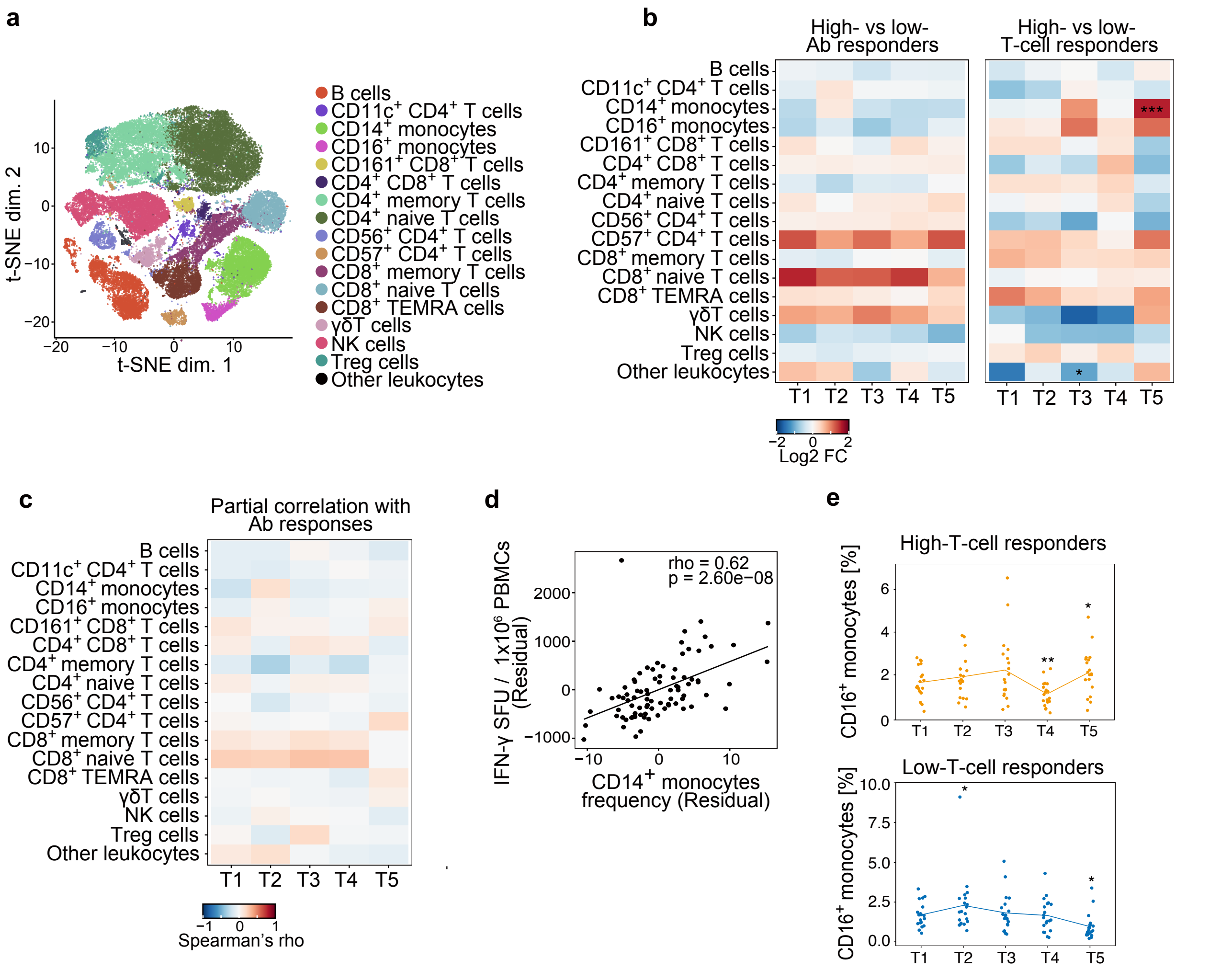

Supplementary Fig. 4. Identification of immune cell populations associated with BNT162b2-induced adaptive immunity by CyTOF analysis.

(a) Immune cell populations in PBMCs isolated from subjects who were baseline seronegative for SARS-CoV-2 spike (n = 86) at time points T1-T5 were analyzed with CyTOF. **(a)** t-distributed stochastic neighbor embedding (t-SNE) visualization of CyTOF data of PBMCs at T1 (n = 86). Types of immune cell populations were annotated based on expression of their marker proteins. TEMRA, terminally differentiated effector memory. NK, natural killer. Treg, regulatory T. **(b)** Heat map showing differences in the frequency of immune cell populations in high- vs low-antibody (Ab) responders or in high- vs low-T-cell responders (n = 10 each) in the discovery cohort. p values were calculated with Wilcoxon signed rank tests with Benjamini–Hochberg FDR correction (*** p < 0.001) **(c)** Heat map showing the correlation between the frequency of immune cell populations and vaccine-induced antibody responses in the entire cohort (n = 86). Partial correlation analyses with adjustments for age and sex were performed with Spearman's correlation tests with Benjamini–Hochberg FDR correction **(d)** Scatterplot showing a correlation between the frequency of CD14^{+} monocytes at T5 and vaccine-induced T-cell responses. **(c-d)** Partial correlation analyses with adjustments for age and sex were performed with Spearman's correlation tests with Benjamini–Hochberg FDR correction. **(e)** Kinetics of the frequency of CD16^{+} monocytes in PBMCs during vaccine response. High-T-cell responders (upper panel, n = 20) and low-T-cell responders (lower panel, n = 20) were analyzed. p values were calculated with Wilcoxon signed rank tests with Benjamini–Hochberg FDR correction (* p < 0.05, ** p < 0.01, *** p < 0.001).

Supplemental Figure 5

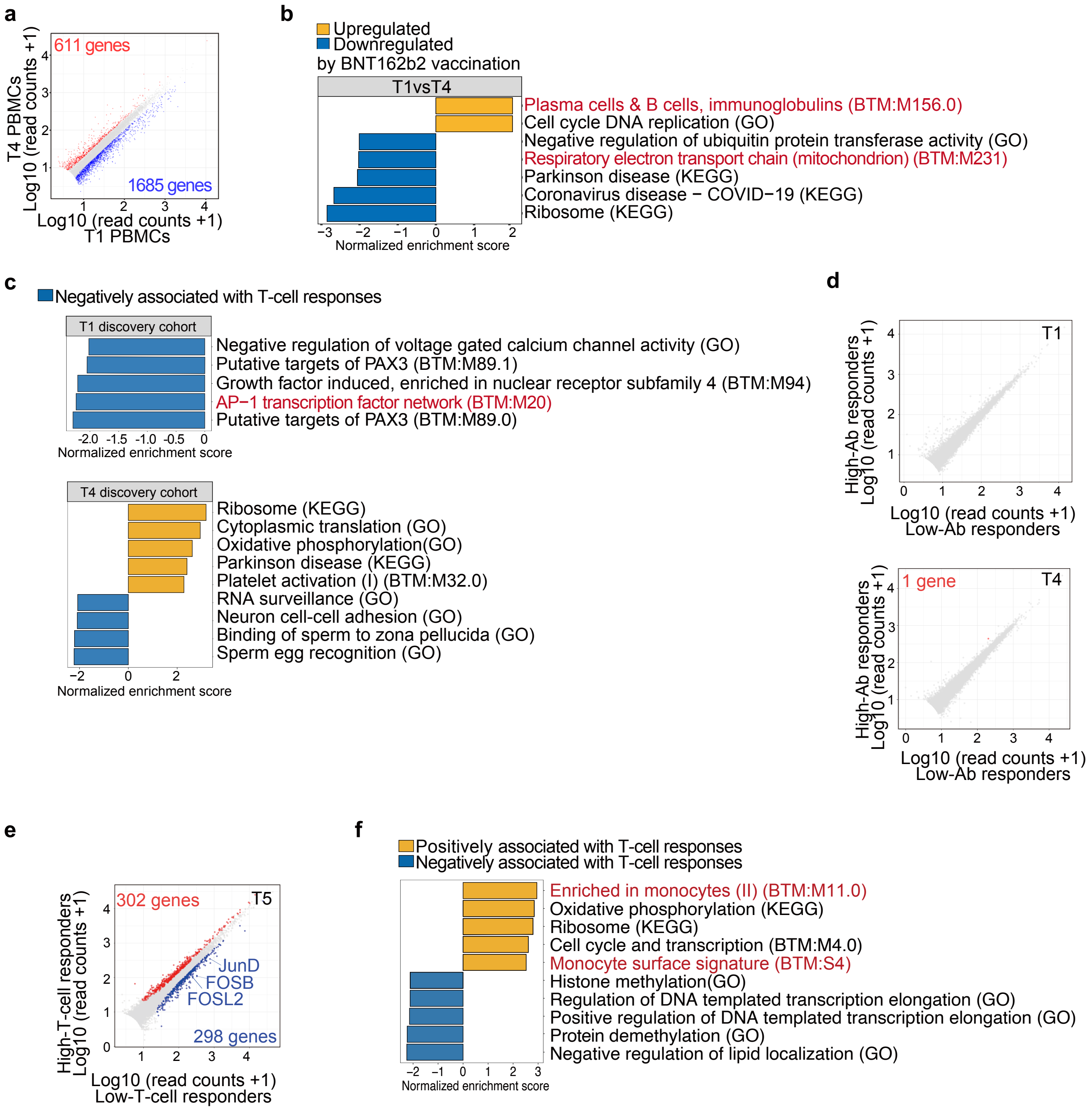

Supplementary Fig. 5. Identification of transcripts associated with BNT162b2-induced adaptive immunity by bulk RNA-seq analysis.

(a) Scatterplots showing DEGs between cells collected at T1 (n =80) and T4 (n = 78) in the entire cohort. **(b)** GSEA on a ranked gene list based on the change in expression between cells collected at T1 and T4 in the entire cohort. **(c)** GSEA on a ranked gene list based on Spearman's correlation coefficients between RNA expression and vaccine-induced T-cell responses (n = 43) in the discovery cohort. **(d)** Scatterplots showing DEGs at T1 or T4 between high- (n = 18 at T1, n = 18 at T4) and low- (n = 19 at T1, n = 20 at T4) antibody responders in the entire cohort. **(e)** Scatterplots showing DEGs at T5 between high- (n = 18) and low- (n = 19) T-cell responders in the entire cohort. **(f)** GSEA on a ranked gene list based on Spearman's correlation coefficient between RNA expression at T5 and vaccine-induced T-cell responses (n = 37: 18 high-T-cell responders and 19 low-T-cell responders). **(a, d, e)** DEGs: differentially expressed genes (log2 FC > 0.5, adjusted p < 0.05). Blue and red dots indicate genes that were highly expressed in the sample groups shown on the X-axis and Y-axis, respectively. N.S.: not significant. **(b-c, f)** Immune-related BTM, GO, and KEGG pathways are shown in red.

Supplementary Figure 6

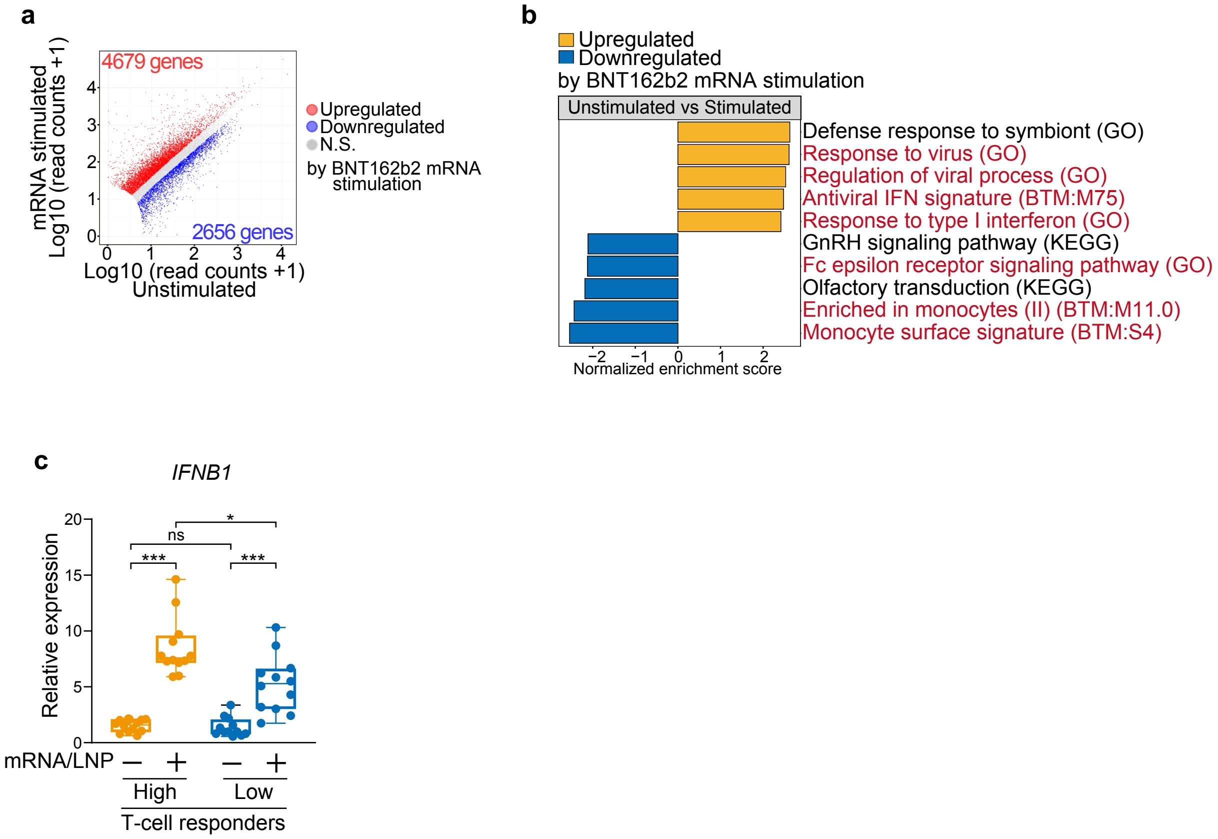

Supplementary Fig. 6. Expression of type I IFN in PBMCs stimulated with BNT162b2 mRNA *ex vivo*.
PBMCs collected from the subjects (n = 86) in the entire cohort at T1 were stimulated with BTN162b2 mRNA for 6 h and analyzed by bulk RNA-seq **(a)** Scatterplot showing DEGs (log2 FC > 0.5, adjusted p < 0.05) between cells unstimulated and stimulated with BNT162b2 mRNA for 6 h. Red and blue dots indicate DEGs that were upregulated and downregulated by BNT162b2 mRNA stimulation, respectively. **(b)** GSEA on a ranked gene list based on the change in expression between cells unstimulated and stimulated with BNT162b2 mRNA. Immune-related BTM, GO, and KEGG pathways are shown in red. **(c)** qPCR analysis of *IFNB1* mRNA expression in PBMCs stimulated with BNT162b2 mRNA encapsulated with LNP for 6 h. The p value was calculated with the Wilcoxon signed rank test (* p < 0.05, *** p < 0.001) with Benjamini–Hochberg FDR correction.

Supplemental Figure 7

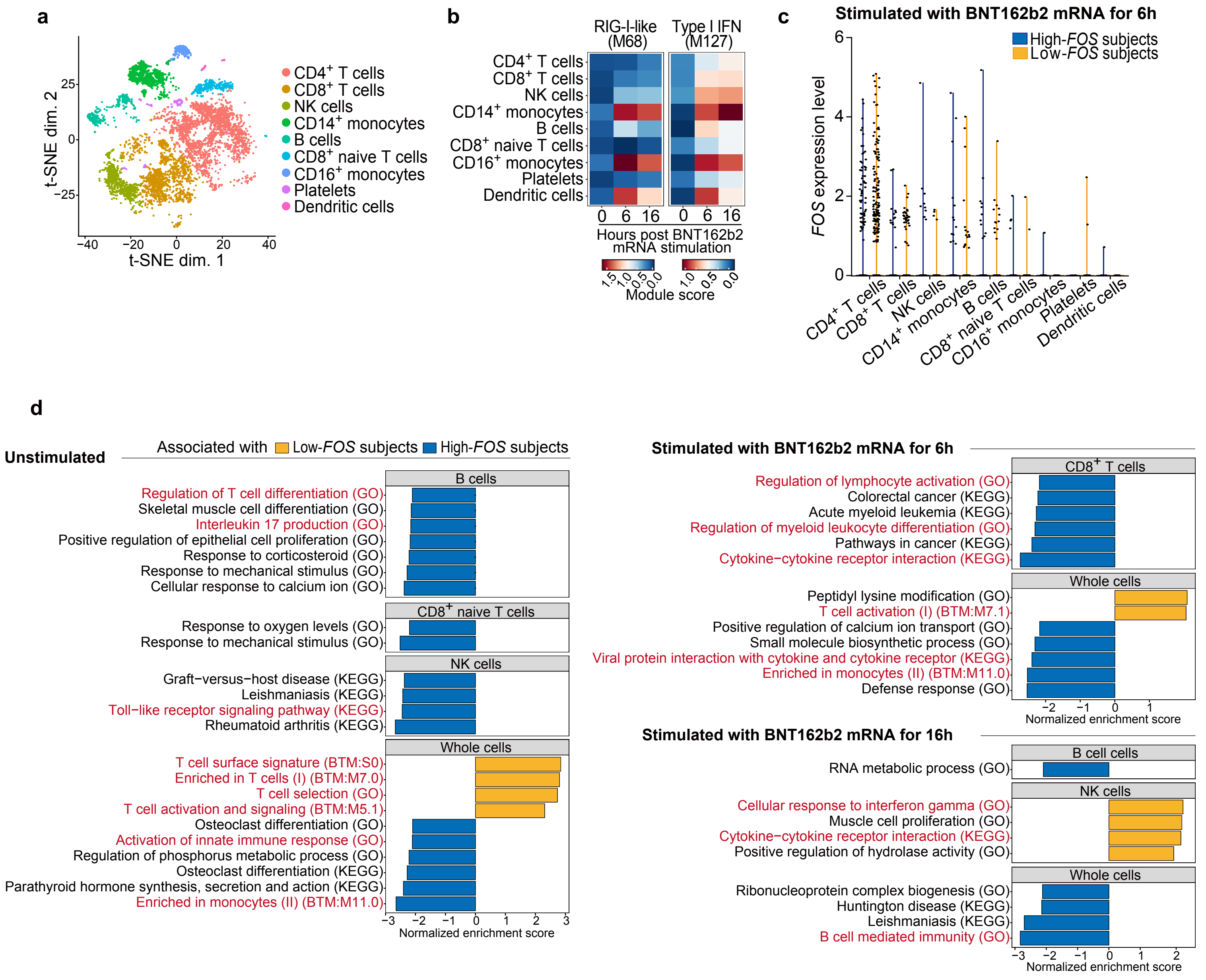

Supplementary Fig. 7. Gene expression profiles in immune cell subpopulations unstimulated or stimulated with BNT162b2 mRNA. scRNA-seq data obtained in Fig. 5 were analyzed. **(a)** t-SNE visualization of scRNA-seq data of unstimulated PBMCs. Data from all eight subjects (high- and low-*FOS* subjects, n = 4 each) were pooled and visualized. **(b)** Module score analysis of genes related to interferon signaling in each immune cell cluster. **(c)** Violin plots showing expression of *FOS* in PBMCs stimulated with BNT162b2 mRNA for 6 h. **(d)** GSEA on a ranked gene list based on the change in expression in various immune cell populations unstimulated or stimulated with BNT162b2 mRNA for 6 or 16 h between high- and low-*FOS* subjects. Immune- related BTM, GO, and KEGG pathways are shown in red.

Supplementary Figure 8

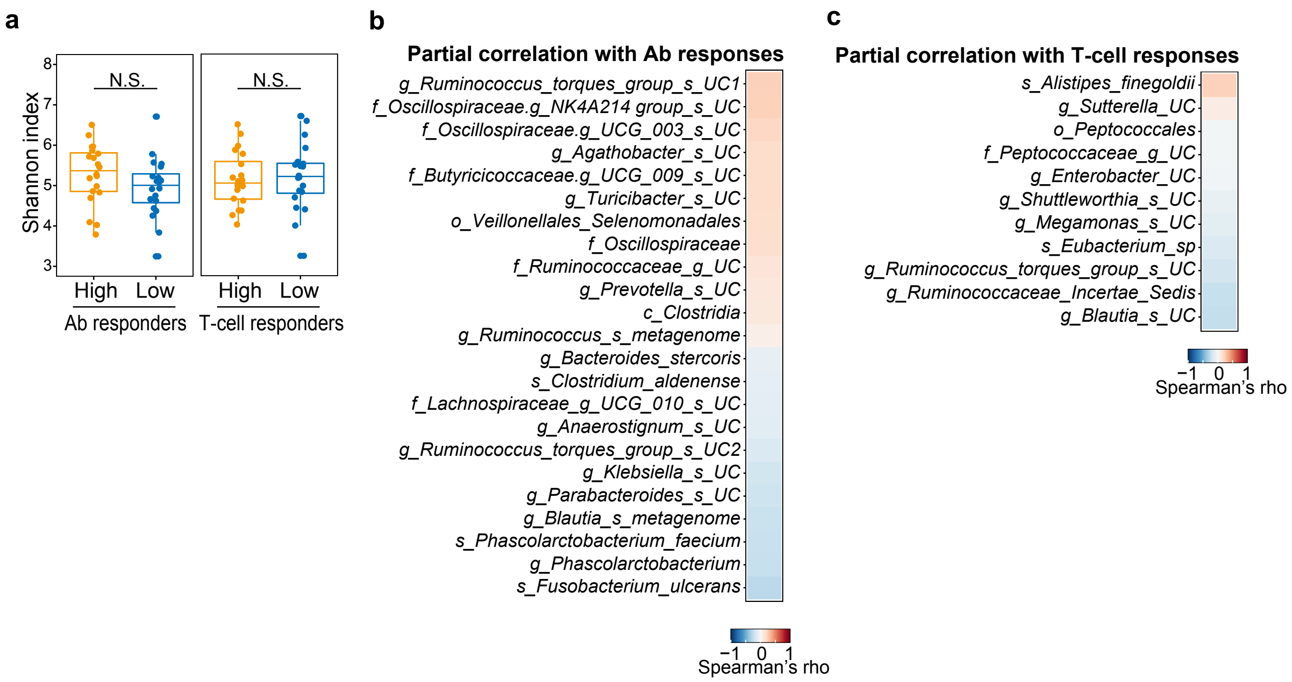

Supplementary Fig. 8. Gut microbial taxa associated with BNT162b2-induced antibody and T cell responses. 16S ribosomal RNA gene sequencing data obtained in Fig. 6 were analyzed. **(a)** Shannon index was compared in high vs low antibody (Ab) responders or in high- vs low-T-cell responders in the entire cohort (n = 86). p values were calculated with Wilcoxon rank-sum tests with Benjamini–Hochberg FDR correction (N.S., not significant). **(b, c)** Heat map showing correlations between specific gut microbes and vaccine-induced antibody **(b)** or T-cell responses **(c)** in the entire cohort (n = 86). Partial correlation analyses with adjustments for age, sex, and stool sampling timing were performed with Spearman's correlation tests with Benjamini–Hochberg FDR correction. There were no significant correlations between the bacterial taxa analyzed and vaccine-induced antibody or T-cell responses. o, order; f, family; g, genus; s, species; UC, unclassified.

Supplementary Figure 9

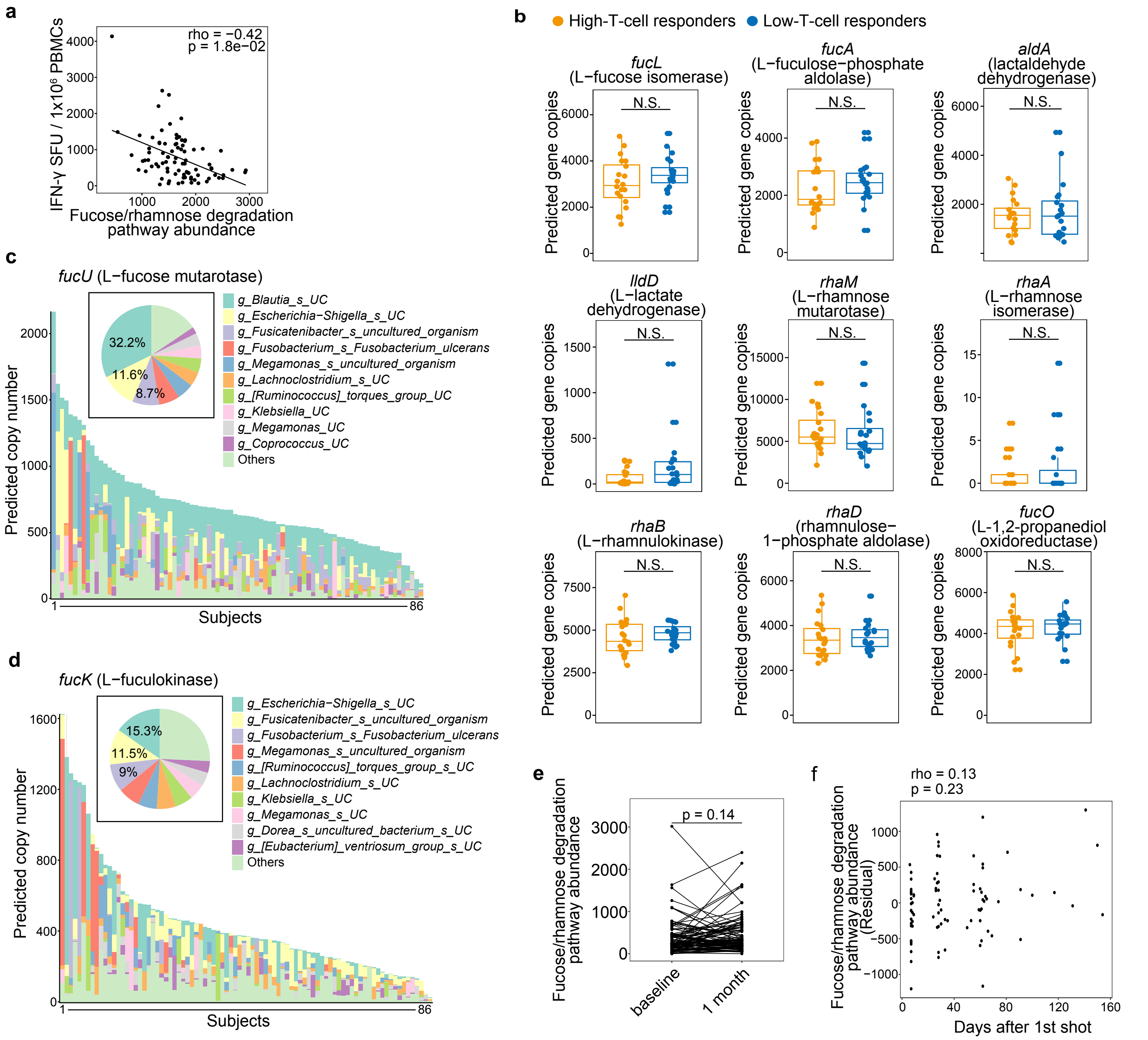

Supplementary Fig. 9. Association between gut microbial fucose/rhamnose degradation and COVID-19 vaccine responses.
(a) Correlations of vaccine-induced T-cell responses with functions of gut microbiota were analyzed with Spearman's correlation tests with Benjamini–Hochberg FDR correction. Scatterplot showing an inverse correlation between the gut microbial fucose/rhamnose degradation pathway and vaccine-induced T-cell responses. **(b)** Analysis of the abundance of predicted copies of genes encoding enzymes involved in the fucose/rhamnose degradation pathway in high- and low-T-cell responders (n = 20 each). p values were calculated with Wilcoxon rank-sum tests with Benjamini–Hochberg FDR correction (N.S., not significant). **(c, d)** The top 10 bacterial taxa that contributed to the abundance of predicted L-fucose mutarotase **(c)** and L-fuculokinase **(d)** genes are shown in pie charts (percentages in the whole subjects) and bar graphs (predicted gene copies in each subject). **(e)** Abundance of genes related to fucose/rhamnose degradation in gut microbiota before and after vaccination. Metagenome data from the European nucleotide archive (ENA, PRJEB35111) were analyzed. **(f)** Correlation analysis between stool sample timing and gut fucose/rhamnose degradation in the entire cohort (n = 86). Partial correlation analysis with adjustments for age and sex was performed with Spearman's correlation tests.

Supplementary Figure 10

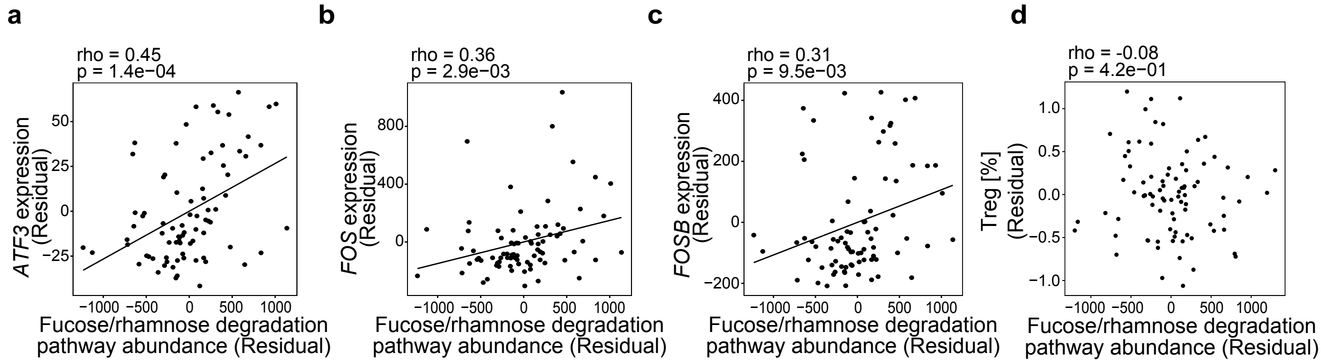

Supplementary Fig. 10. Gut microbial fucose/rhamnose degradation is associated with baseline AP-1 expression but not with frequency of Treg cells in PBMCs.

(a-c) Scatterplots showing the correlation between the gut microbial fucose/rhamnose degradation pathway and baseline expression of *ATF3* **(a)**, *FOS* **(b)**, and *FOSB* **(c)** in the entire cohort (n = 86). **(d)** Using the entire cohort data (n = 86), the correlation between fucose/rhamnose degradation and the frequency of Treg cells was analyzed. Partial correlation analysis with adjustments for age, sex, and stool sampling timing was performed with Spearman's correlation tests.

Supplementary Table1. Sequencing metrics of scRNAseq

	# of cells loaded	# of cells recovered	multiplet rate
**Baseline**	20000	12134	39%
**6h after *ex vivo* stim.**	20000	12239	37%
**16h after *ex vivo* stim.**	20000	11587	33%

Supplementary Table2. List of primer sets

Name	Sequence
*ACTB*_F	ACAGAGCCTCGCCTTTG
*ACTB*_R	CCTTGCACATGCCGGAG
*IFNB1*_F	AAACTCATGAGCAGTCTGCA
*IFNB1*_R	AGGAGATCTTCAGTTTCGGAGG
*PTGS2*_F	CGGTGAAACTCTGGCTAGACAG
*PTGS2*_R	GCAAACCGTAGATGCTCAGGGA
*FOS*_F	GCCTCTCTTACTACCACTCACC
*FOS*_R	AGATGGCAGTGACCGTGGGAAT
