# Supplementary material for: Human immune and gut microbial parameters associated with inter-individual variations in COVID-19 mRNA vaccine-induced immunity
Source: Commun Biol. 2023 Apr 20;6:368. doi: 10.1038/s42003-023-04755-9 (PMC10119155; doi:10.1038/s42003-023-04755-9)
Supplement: Supplementary file 5 — Reporting Summary [file 42003_2023_4755_MOESM5_ESM.pdf]

nature portfolio

Corresponding author(s): Hiroki Ishikawa

Last updated by author(s): 2023/3/14

Reporting Summary

Nature Portfolio wishes to improve the reproducibility of the work that we publish. This form provides structure for consistency and transparency in reporting. For further information on Nature Portfolio policies, see our Editorial Policies and the Editorial Policy Checklist.

Statistics

For all statistical analyses, confirm that the following items are present in the figure legend, table legend, main text, or Methods section.

n/a	Confirmed	
☐	☒	The exact sample size (*n*) for each experimental group/condition, given as a discrete number and unit of measurement
☐	☒	A statement on whether measurements were taken from distinct samples or whether the same sample was measured repeatedly
☐	☒	The statistical test(s) used AND whether they are one- or two-sided<br>*Only common tests should be described solely by name; describe more complex techniques in the Methods section.*
☐	☒	A description of all covariates tested
☐	☒	A description of any assumptions or corrections, such as tests of normality and adjustment for multiple comparisons
☐	☒	A full description of the statistical parameters including central tendency (e.g. means) or other basic estimates (e.g. regression coefficient) AND variation (e.g. standard deviation) or associated estimates of uncertainty (e.g. confidence intervals)
☐	☒	For null hypothesis testing, the test statistic (e.g. *F*, *t*, *r*) with confidence intervals, effect sizes, degrees of freedom and *P* value noted<br>*Give P values as exact values whenever suitable.*
☒	☐	For Bayesian analysis, information on the choice of priors and Markov chain Monte Carlo settings
☒	☐	For hierarchical and complex designs, identification of the appropriate level for tests and full reporting of outcomes
☐	☒	Estimates of effect sizes (e.g. Cohen's *d*, Pearson's *r*), indicating how they were calculated

Our web collection on statistics for biologists contains articles on many of the points above.

Software and code

Policy information about availability of computer code

Data collection	No software was used to collect data
Data analysis	Cytobank (v.9.1), cyCombine (v.0.1.8), CATALYST (v.3.16), flowCore (v.2.6.0), fastPG (v.0.0.8), FastQC (v.0.11.9), Trimmomatic (v.0.39), HISAT2 (v.2.2), GENCODE (v.30), Subread (v.2.0.1), DESeq2 (v.1.34.0), clusterProfiler(v.4.2.2), iRegulon (v.1.3), Cytoscape (v.3.9.1), SAMtools (v.1.12), BEDtools (v.2.26.0), BCFtools (v.1.11-1), VCFtools (v.0.1.16-2), CellRanger Single-Cell Software Suite (v.6.0.0), demuxlet (v.2.0.1), Seurat(v.4.1.0), QIIME2 (v.2020.2), PICRUSt2 (v.2.4.2), R (v.4.1.2), GraphPad Prism (v.9.4.0)

For manuscripts utilizing custom algorithms or software that are central to the research but not yet described in published literature, software must be made available to editors and reviewers. We strongly encourage code deposition in a community repository (e.g. GitHub). See the Nature Portfolio guidelines for submitting code & software for further information.

Data

Policy information about availability of data

All manuscripts must include a data availability statement. This statement should provide the following information, where applicable:

- Accession codes, unique identifiers, or web links for publicly available datasets
- A description of any restrictions on data availability
- For clinical datasets or third party data, please ensure that the statement adheres to our policy

scRNA-seq, bulk RNA-seq, and 16S rRNA gene sequencing data that support the finding of this study have been deposited to DDBJ database under accession numbers DRA014613, DRA014614, and DRA014615, respectively. Any other relevant data are available from the corresponding author upon request.

Human research participants

Policy information about studies involving human research participants and Sex and Gender in Research.

Reporting on sex and gender	Ninety-six Japanese healthy volunteers (42 men and 53 women; average age, 52.4±14.9years; age range: 20–81years) were recruited in Okinawa, Japan, between May 2021 and August 2021. All participants provided informed written consent. Sex was determined based on self-reporting.
Population characteristics	see above
Recruitment	see above
Ethics oversight	The study was approved by the Okinawa Institute of Science and Technology, Graduate University (OIST) human subjects ethics committee (application HSR-2021–001). All participants provided informed written consent.

Note that full information on the approval of the study protocol must also be provided in the manuscript.

Field-specific reporting

Please select the one below that is the best fit for your research. If you are not sure, read the appropriate sections before making your selection.

☒ Life sciences ☐ Behavioural & social sciences ☐ Ecological, evolutionary & environmental sciences

For a reference copy of the document with all sections, see nature.com/documents/nr-reporting-summary-flat.pdf

Life sciences study design

All studies must disclose on these points even when the disclosure is negative.

Sample size	Sample sizes were determined by availability of data
Data exclusions	data from one participant who was not able to receive the second dose in a timely manner due to severe side effects from the first dose were excluded from the analysis
Replication	all attempts at replication were successful
Randomization	86 subjects seronegative for SARS-CoV-2 spike at baseline (hereafter referred to as the entire cohort) were randomly divided into discovery and validation cohorts (n = 43 each)
Blinding	Blinding is not relevant to our study since

Reporting for specific materials, systems and methods

We require information from authors about some types of materials, experimental systems and methods used in many studies. Here, indicate whether each material, system or method listed is relevant to your study. If you are not sure if a list item applies to your research, read the appropriate section before selecting a response.

Materials & experimental systems

n/a	Involved in the study	
☐	☒	Antibodies
☒	☐	Eukaryotic cell lines
☒	☐	Palaeontology and archaeology
☒	☐	Animals and other organisms
☒	☐	Clinical data
☒	☐	Dual use research of concern

Methods

n/a	Involved in the study	
☒	☐	ChIP-seq
☒	☐	Flow cytometry
☒	☐	MRI-based neuroimaging

Antibodies

Antibodies used

For CyTOF analysis, following antibodies were used: CD45-89Y, CD196/CCR6-141Pr, CD123-143Nd, CD19-144Nd, CD4-145Nd, CD8a-146Nd, CD11c-147Sm, CD16-148Nd, CD45RO-149Sm, CD45RA-150Nd, CD161-151Eu, CD194/CCR4-152Sm, CD25 -153Eu, CD27-154Sm, CD57-155Gd, CD183/CXCR3-156Gd, CD185/CXCR5-158Gd, CD28- 160Gd, CD38- 161Dy, CD56/NCAM- 163Dy, TCRgd-164Dy, CD294-166Er, CD197/CCR7-167Er, CD14-168Er, CD3-170Er, CD20-171Yb, CD66b-172Yb, HLA-DR-173Yb, IgD-174Yb, CD127- 176Yb, Live/dead intercalator-103Rh. All antibodies were obtained from Fluidigm.

Validation

Antibody validation was as described by manufacturer, Fluidigm.
